# Tal1_NXtc01_ in *Xanthomonas translucens* pv. *cerealis* Contributes to Virulence in Bacterial Leaf Streak of Wheat

**DOI:** 10.3389/fmicb.2019.02040

**Published:** 2019-09-04

**Authors:** Syed Mashab Ali Shah, Fazal Haq, Wenxiu Ma, Xiameng Xu, Sai Wang, Zhengyin Xu, Lifang Zou, Bo Zhu, Gongyou Chen

**Affiliations:** School of Agriculture and Biology/State Key Laboratory of Microbial Metabolism, Shanghai Jiao Tong University, Shanghai, China

**Keywords:** bacterial leaf streak, *Xanthomonas translucens* pv. *cerealis*, SMRT sequencing, TALE, T3SS, virulence

## Abstract

*Xanthomonas translucens* pv. *cerealis* (*Xtc*) causes bacterial leaf streak (BLS) of important cereal crops, including wheat (*Triticum aestivum*) and barley (*Hordeum vulgare*). Transcription activator-like effectors (TALEs) play vital roles in many plant diseases caused by *Xanthomonas* spp., however, TALEs have not been previously characterized in *Xtc*. In this study, the whole genome of NXtc01, a virulent strain of *Xtc* from Xinjiang, China, was sequenced and compared with genomes of other *Xanthomonas* spp. *Xtc* NXtc01 consists of a single 4,622,298 bp chromosome that encodes 4,004 genes. Alignment of the NXtc01 sequence with the draft genome of *Xtc* strain CFBP 2541 (United States) revealed a single giant inversion and differences in the location of two *tal* genes, which were designated *tal1* and *tal2*. In NXtc01, both *tal* genes are located on the chromosome, whereas *tal2* is plasmid-encoded in CFBP 2541. The repeat variable diresidues (RVDs) at the 12th and 13th sites within Tal2 repeat units were identical in both strains, whereas Tal1 showed differences in the third RVD. *Xtc* NXtc01 and CFBP 2541 encoded 35 and 33 non-TALE type III effectors (T3Es), respectively. *tal1*, *tal2*, and *tal*-free deletion mutants of *Xtc* NXtc01 were constructed and evaluated for virulence. The *tal1* and *tal*-free deletion mutants were impaired with respect to symptom development and growth in wheat, suggesting that *tal1* is a virulence factor in NXtc01. This was confirmed in gain-of-function experiments that showed the introduction of *tal1*, but not *tal2*, restored virulence to the *tal*-free mutant. Furthermore, we generated a *hrcC* deletion mutant of NXtc01; the *hrcC* mutant was non-pathogenic on wheat and unable to elicit a hypersensitive response in the non-host *Nicotiana benthamiana*. Our data provide a platform for exploring the roles of both TALEs and non-TALEs in promoting BLS on wheat.

## Introduction

*Xanthomonas* is a large genus of plant-associated, Gram-negative bacteria and contains 27 species that cause disease on approximately 124 monocots and 268 dicots (Chan and Goodwin, 1999). *Xanthomonas* spp. are subdivided into pathovars on the basis of host specificity and can also be divided into two main phylogenetic groups based on sequence analysis of 16S rDNA, *gyrB, dnaK*, *rpoD*, and *fyuA* (Hauben et al., 1997; Young et al., 2008). The smaller Group 1 includes diverse *Xanthomonas* spp. including *X. translucens*, *X. albilineans*, *X. hyacinthi*, *X. theicola* and *X. sacchari*, whereas the larger Group 2 contains *X. oryzae, X. campestris, X. vasicola, X. axonopodis, X. euvesicatoria*, and *X. citri.* Some species are comprised of numerous pathovars (Parkinson et al., 2007); for example, *X. translucens* includes *X. translucens* pv. *cerealis* (*Xtc*), *X. translucens* pv. *translucens* (*Xtt*), and *X. translucens* pv. *undulosa* (*Xtu*).

*X. translucens* causes disease in a wide variety of economically-important crops. An emerging disease of global importance is bacterial leaf streak (BLS), which is caused by *Xtc*, *Xtt* and *Xtu* (Pesce et al., 2015; Jaenicke et al., 2016; Peng et al., 2016; Falahi Charkhabi et al., 2017). *Xtc* has a broad host range with respect to cereals and infects wheat (*Triticum spp.*), triticale (*Triticosecale*), barley (*Hordeum vulgare*), rye (*Secale cereale*), and oats (*Avena* spp.) (Cunfer and Scolari, 1982; Bragard et al., 1997; Adhikari et al., 2012); symptoms are particularly severe on wheat (Adhikari et al., 2012). Reported yield losses due to BLS in cereals range from 8 to 34% depending on host susceptibility and the amount of inoculum, while the highest documented yield loss is 40% (Shane et al., 1987; Forster and Schaad, 1988; Duveiller and Maraite, 1993). BLS can also impact grain quality when it occurs on spikes (Mehta, 1990).

*Xanthomonas* pathogens use similar strategies to release virulence factors into the host including the production of exopolysaccharides (EPS), cell wall-degrading enzymes and protein secretion systems (Büttner and Bonas, 2010). *Xanthomonas* spp. produce type III effectors (T3Es) that can modulate virulence in host plants; T3Es are delivered into plant cells via the type III secretion system (T3SS), which is encoded by the *hrp* [hypersensitive response (HR) and pathogenicity] regulon (White et al., 2009). This *hrp* regulon is positioned on approximately 20 kb genomic island that encode more than 20 *hrp* genes in many operons. Individual genes have been classified as *hrp*, *hrc* (*hrp* conserved) and *hpa* (*hrp* associated). Originally, the “*hrp*” and “*hrc*” designations indicated loci that are required for non-host hypersensitive reaction and pathogenicity, but not all of them have this phenotype. *hrc* genes sequences are clearly conserved among animal and plant pathogens, while the sequences of *hrp* genes are unique among *Xanthomonas* and some other genera with some exceptions (Bogdanove et al., 1996). The *hpa* genes have their role in pathogenicity, depending on the gene. Some function in targeting the type-III secreted proteins to the secretion apparatus, some are themselves translocated into host cells, and some have unknown roles (Kim et al., 2003; Lorenz et al., 2008).

In phytopathogenic bacteria *hrp* gene clusters are classified into two groups, based on the repertoire genes organization and regulation. Group I includes *Pseudomonas syringae*, *Erwinia* spp., and *Pantoea stewartii* subsp. *stewartii* while group II comprises *Ralstonia solanacearum*, *Acidovorax avenae*, and *Xanthomonas* spp. (Alfano and Collmer, 1997). The expression of group II *hrp* genes are controlled by two key regulatory genes, *hrpG* and *hrpX* (syn. *hrpB*) (Büttner and Bonas, 2010). In *Xanthomonas* the *hrp* gene cluster was previously known as a pathogenicity island (Noël et al., 2002) but it is reported missing from some *Xanthomonas* spp. The largest and well-studied *Xanthomonas* group, Group 2 contain a canonical *hrp* gene clusters but some studies demonstrated the absence of Hrp T3SS from the group 2 strains that are pathogenic on cannabis and barley (Ignatov et al., 2015; Jacobs et al., 2015). The diverse and smaller group of *Xanthomonas*, Group 1, contains Hrp T3SS with the exceptions of *X. albilneans* and *X. sacchari* (Pieretti et al., 2009; Studholme et al., 2011), all others revealed Hrp T3SS but differing degree of genetic organization to those from group 2 *Xanthomonas* (Pesce et al., 2017). In *X. translucens* the mutants of *hrp* structural genes *hrcC* of *Xtu* strain XT4699 pathogenic on various members of *Poaceae* were impaired in causing symptoms (Peng et al., 2016) while mutants of *hrcR*, *hrpE*, and *hrpG* of *Xtg*29 pathogenic on forage grass reduced disease symptoms but multiplication of mutants was not affected relative to wild type (Wichmann et al., 2013).

Hrp T3SS is involved in the translocation of type III secreted effector proteins that play a key role in complex process of pathogenesis and host adaptation (Büttner and Bonas, 2010). These T3Es are categorized into transcription activator-like effectors (TALEs) and non-TALEs, which are commonly known as *Xanthomonas* outer proteins or Xops. The TALE proteins localize to the host plant nucleus, where they recognize and activate effector-binding elements (EBEs) that reside in the promoter regions of host susceptibility (*S*) or resistance (*R*) genes. Common structural features of TALEs include an N-terminal domain that harbors the type III secretion signal, a central repeat region (CRR) that interacts with host EBEs, a C-terminal domain that contains nuclear localization signals, and an acidic activation domain. The CRR encodes tandem repeats of 33–35 amino acids and residues at positions 12 and 13 are known as repeat-variable diresidues (RVDs); residue 13 is responsible for base-specific DNA targeting (Boch and Bonas, 2010; Mak et al., 2013; Ji et al., 2016). Evolutionary modifications occur in TALEs by substitution, recombination and deletion events in the nucleotide sequence, but individual RVDs are highly conserved (Erkes et al., 2017). TALE-like proteins can occur in other organisms; examples include the RipTALEs in *Ralstonia solanacearum*, the MOrTL proteins in marine microorganisms and the *Burkholderia rhizoxinica* Bat proteins (de Lange et al., 2015).

The number of *tal* genes in *Xanthomonas* spp. is highly variable; for example, strains of *X. translucens* pv. *translucens*, pv. *undulosa*, and pv. *cerealis* were shown to harbor eight, seven and two *tal* genes, respectively (Pesce et al., 2015; Jaenicke et al., 2016; Peng et al., 2016; Falahi Charkhabi et al., 2017). Some TALEs function to induce plant host *S* genes, and this fosters pathogen growth and disease development. For example, *X. oryzae* pv. *oryzae* (*Xoo*), which causes bacterial leaf blight (BLB) in rice, targets the host *S* genes *OsSWEET11* via TALE PthXo1, *OsSWEET13* via PthXo2 and *OsSWEET14* by TALEs AvrXa7, Tal5, TalC and PthXo3 (Yang et al., 2006; Streubel et al., 2013; Zhou et al., 2015). In the BLS pathogen *X. oryzae* pv. *oryzicola* (*Xoc*), the Tal7 effector activates the rice gene *Os09g29100*, which suppresses the host immune response (Cai et al., 2017). TALEs can also induce the expression of plant transcription factors; for example, PthXo6 and PthXo7 induce *OsTFX1* and *OsTFIIA*γ*1*, respectively (Sugio et al., 2007; Ma et al., 2018). Similarly, AvrHah1 of *X. gardneri* activates the expression of two bHLH (basic helix-loop-helix) transcription factors (Schwartz et al., 2017).

Some TALEs activate host resistance (*R*) genes that restrict bacterial growth and disease development through rapid cell death. For example, the pepper *R* gene, *Bs3*, is activated by AvrBs3 in *X. euvesicatoria*, and rice *R* genes *Xa10*, *Xa23*, and *Xa27* are activated by AvrXa10, AvrXa23, and AvrXa27 in *Xoo* (Gu et al., 2005; Römer et al., 2007; Ji et al., 2014; Tian et al., 2014; Wang et al., 2015). Individual TALEs can also deploy disease resistance through direct protein-protein interaction rather than targeting host *R* or *S* genes (Read et al., 2016). Overall, it is well-accepted that TALE proteins in pathogens and their targets in host plants are engaged in an ongoing, co-evolutionary arms race (Ji et al., 2016).

Although several *X. translucens* strains genome sequences are available and revealed many shared virulence factors (Wichmann et al., 2013; Pesce et al., 2015; Hersemann et al., 2016; Jaenicke et al., 2016; Peng et al., 2016; Falahi Charkhabi et al., 2017; Langlois et al., 2017). Among them, TALEs play important role in plant diseases. Recently, a complete genome sequence of highly virulent Iranian strain *Xtu* ICMP11055 revealing seven TALE genes. Indel mutagenesis and complementation analysis revealed that two TALE genes, Tal2 and Tal4b contribute to the disease development (Falahi Charkhabi et al., 2017), no other *X. translucens* pathovars TALEs are reported that contribute to virulence up-to-date.

Bacterial leaf streak of wheat, which is caused by *Xtc*, is an important, emerging disease that has led to significant yield losses in Xinjiang Province, China. *Xtc* NXtc01 is a virulent strain isolated from wheat cultivated in Xinjiang. To better understand the mechanistic basis of pathogenesis in the *Xtc*/wheat interaction, we sequenced the whole genome of *Xtc* using SMRT technology and compared it with the genomes of *X. translucen*s, *X. campestris*, and *X. oryzae*. We also compared NXtc01 genes encoding T3SS gene cluster and T3Es, particularly TALEs, with homologs in other *Xanthomonas* spp. Two *tal* genes were deleted in NXtc01, and their role in *Xtc* virulence was investigated.

## Materials and Methods

### Bacterial Strains, Growth Conditions, Plasmids and Primers

The bacterial strains, plasmids and primers used in this study are provided in Supplementary Tables S1, S2. *X. translucens* pv. *cerealis* strains were cultured in NB medium at 28^∘^C, and *Escherichia coli* was grown in LB medium at 37^∘^C (Miller, 1972). Plasmids were transferred into *Xtc* NXtc01 and *E. coli* DH5α by electroporation and heat shock, respectively (Hanahan et al., 1991). Antibiotics used for screening are as follows (μg ml^–1^): kanamycin (Km), 20; ampicillin (Ap), 100; and spectinomycin (Sp), 25.

### Genomic and Plasmid DNA Extraction

All bacterial strains were stored at −80^∘^C in 50% glycerol. The NXtc01 genomic DNA used for PacBio sequencing was extracted from a 24 h culture using the Hipure bacterial DNA kit (Magen, Guangzhou, Guangdong, China) as recommended by the manufacturer. Genomic DNA was isolated from the *Xtc* mutant strains using the same method and prepared for Southern blotting (see below). DNA quality and quantity were checked with a NanoDrop spectrophotometer and screened for plasmids by agarose gel electrophoresis (Chakrabarty et al., 2010). Plasmids were isolated from *E. coli* DH5α using the Plasmid DNA Mini Kit (GBS Biotechnology, China).

### PacBio Sequencing, Assembly and Annotation

**FIGURE 1:**
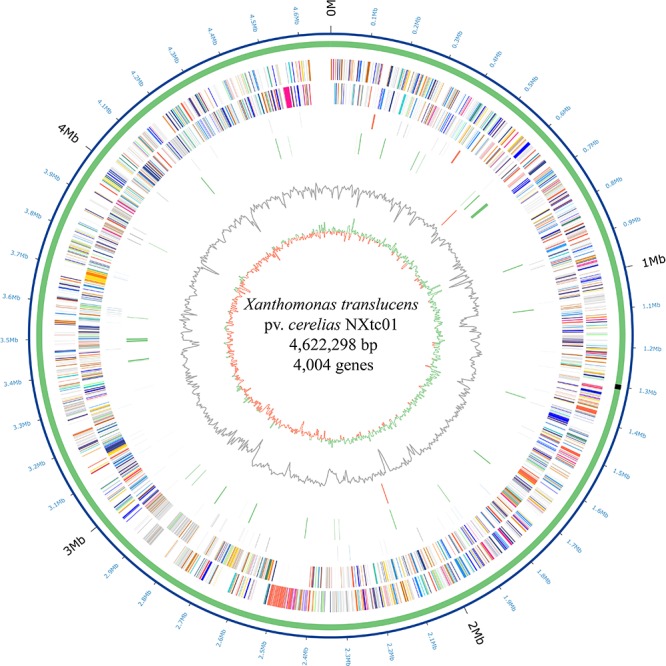
Circular representation of the *X. translucens* pv. *cerealis* strain NXtc01 genome starting from *gyrB*. Inner to outermost rings illustrate GC skew (G − C)/(G + C), GC contents, *tal* genes (orange), IS elements (green), tRNA (blue), rRNA (orange) and ncRNA (black) genes, reverse COGs (cluster of orthologous groups), forward COGs (Supplementary Table S4), chromosome (green), and coordinates (Mb). The T3SS genes cluster is indicated as black rectangle on chromosome.

The NXtc01 genome was sequenced on a PacBio RSII platform (Pacific Biosciences) using Single Molecule, Real Time (SMRT) DNA sequencing. Totally 304,280 reads (2,107,977,050 total reads base) were obtained with average length 6,927 bp and 287.72x coverage after filtering data through SMRT analysis software v2.3. The genome assembly was done using *de novo* assembly, HGAP protocol available with SMRT analysis packages and can be accessed at SMRT Analysis portal v2.1. The verification of assembly was performed on CheckM with default parameters (Parks et al., 2015). The genome was annotated using the NCBI Prokaryotic Genome Annotation Pipeline (PGAP) and deposited in GenBank as accession number PRJNA528834. The Circos was used to generate the circular genome map of NXtc01 including all the predicted ORFs (Krzywinski et al., 2009) as shown in Figure 1.

### Construction of Phylogenetic Trees and Tal RVDs Comparison

A total of 12 strains were selected for homology searches. Ortho MCL v. 2.0 was used to generate groups of orthologous proteins with default parameters^1^. The resulting set of 1,270 single copy genes was used selected to construct a phylogenetic tree as described previously by Zhu et al. (2011).

In addition to the *Xtc* NXtc01, all available *X. translucens* genome sequences were obtained from the NCBI and used for TALEs phylogenetic analysis. TALE genes were predicted and analyzed in each genome using AnnoTALE v1.2 (Grau et al., 2016). DisTAL v1.1 was used to align and phylogenetically classify TAL effectors based on their repeat arrangement (Pérez-Quintero et al., 2015).

For the analysis of TALEs RVDs, we used AnnoTALE version 1.2 that provides 516 TALE genes of 33 *Xanthomonas* strains. First we analyzed all the *X. translucens* genomes and merged the TALEs RVDs output file into available 516 TALEs RVDs. Finally, we grouped the TALEs into classes on the basis of RVDs that indicates possible functional and evolutionary relationship (Grau et al., 2016; Erkes et al., 2017).

### Identification of Genes Encoding the T3SS and T3Es

Genes encoding the T3SS and effectors (T3Es) were identified in the NXtc01 genome using BLASTn and BLASTp as described by Peng et al. (2016) and Falahi Charkhabi et al. (2017).

### Cloning and Expression of NXtc01 *tal* Genes

NXtc01 *Bam*HI-digested genomic DNA (50 μg) was subjected to 1.2% agarose gel electrophoresis as described by Cernadas et al. (2014). Regions containing *tal* genes (*tal1*, 2,745 bp; *tal2*, 3,872 bp) were gel-purified and ligated into *Bam*HI-linearized and CIP (calf intestinal phosphatase)-treated pBluescript II. Ligated products were transformed into DH5α by heat shock. *Tal-*containing clones were confirmed by colony blots, restriction digestion and Sanger DNA sequencing.

For expression in NXtc01, pBluescript II clones containing *tal* genes were digested at *Sph*I sites to release the CRR and ligated into *Sph*I-linearized, CIP-treated pZW vector. This vector contains a FLAG-tagged derivative of *avrXa10* with the N- and C-terminal domains of the effector (without the CRR), a strong *lac* promoter, and transcription termination signals (Yang et al., 2000). Ligated products were used to transform DH5α; the orientation of *tal* genes was then analyzed by PCR using primers TALN18S-F/TalN-R (Supplementary Table S2) and confirmed by sequence analysis. For expression in *Xanthomonas*, pZW vectors harboring *tal* genes were ligated into pHMI at the *Hin*dIII site and transferred into the *tal*-free strain; these were designated as PHZW-*tal1* and PHZW-*tal2*. To confirm of *tal* gene expression, bacterial cells containing the C-terminally flag-tagged *tal* genes (PHZW-*tal*) were harvested and washed in phosphate-buffered saline (PBS). Cells were harvested by centrifugation, diluted in PBS and boiled for 8 min. Proteins were separated on 8% SDS-PAGE gels and transferred to polyvinylidene difluoride membranes for immunoblotting using mouse anti-flag as the primary antibody (Transgene, Beijing, China). Primary antibodies were detected and visualized using goat anti-mouse IgG (H + L) and the EasySee Western Kit (Transgene) (Supplementary Figure S1C).

### Mutagenesis of NXtc01 *tal* and *hrcC* Genes

The *tal1*, *tal2*, *tal*-free, and *hrcC* mutants were generated by homologous recombination using suicide vector pKMS1 (Zou et al., 2011). For *tal1*, unique 640 bp upstream and 425 bp downstream sequences flanking *tal1* gene were amplified using primer sets T1Nt-F/T1Nt-R and T1Ct-F/T1Ct-R (Supplementary Table S2), respectively. The primers contained *Xba*I (T1Nt-R and T1Ct-F), *Sma*I (T1Ct-R), and *Pst*I (T1Nt-F) sites. The two PCR products were gel-purified, digested with *Xba*I and ligated at 22^∘^C for 2 h. The ligation of upstream and downstream fragments was confirmed by PCR using primer set T1Nt-F/T1Ct-R. The desired fragment was gel-purified, digested with *Sma*I and *Pst*I and cloned into pKMS1. The ligation mixture was introduced into DH5α cells and cultured on LB containing Km. The resulting construct, pKMSTal1 (Supplementary Table S1), was confirmed by colony PCR using a M13 universal primer set and sequenced. pKMSTal1 was introduced into *Xtc* NXtc01, plated on NAN containing Km (NAN^Km^), and incubated for 4 days. NAN^Km^ colonies were then cultured overnight on NAS (NA containing 10% sucrose) and NAN/NAN^Km^, individually. Colonies that grew only on NAN/NAN^Km^ were then transferred to NAN broth for 10 h at 28^∘^C (∼OD_600_ ≤ 0.1). The culture was plated on NAS for 2 days and colonies were transferred to NA and NA^Km^. Colonies that did not grow on NA^Km^ were analyzed by PCR with primer set T1Nt-F/T1Ct-R (Supplementary Table S2 and Supplementary Figure S1A) and were further confirmed by Southern blot analysis (Supplementary Figure S1B).

Similar to the strategy mentioned above, pKMSTal2 was constructed by cloning fragments flanking the CRR in *tal2* (upstream fragment, 698 bp; downstream, 413 bp) into pKMS1 using primer sets T2Ct-F/T2Ct-R and T2Nt-F/T2Nt-R (Supplementary Tables S1, S2 and Supplementary Figure S1A). Construct pKMShrcC was constructed by cloning fragments that flank the *hrcC* gene (upstream fragment, 610 bp; downstream, 694 bp) using primer sets *hrcC*-up-F/*hrcC*-up-R and *hrcC*-do-F/*hrcC*-do-R (Supplementary Tables S1, S2 and Supplementary Figure S1A). A *tal*-free mutant was generated by transferring pKMSTal2 into the *Xtc* NXtc01 *tal1* mutant (Δ*tal1*) following the same strategy described above (Supplementary Tables S1, S2 and Supplementary Figure S1A). The *tal2*, *hrcC*, and *tal*-free mutants were generated and screened using the protocol described for the *tal1* mutant.

### Southern Blot Analysis

Genomic DNA was extracted from NXtc01 and *tal* mutants and digested with *Bam*HI for 5 h. Digested DNA fragments were separated in 1.3% agarose gels in TAE buffer at 4^∘^C, 80 V for 14–16 h and then transferred to Hybond N^+^ nylon membranes (Pall Corporation, NY, United States). The *Sph*I fragment of the NXtc01 *tal1* gene was used as a probe and labeled with digoxigenin (DIG) using the Dig-High Prime Labeling and Detection kit (Roche, Germany). DIG labeling, hybridization, washing, blocking, and detection were conducted as recommended by the manufacturer.

### Plant Inoculation and Quantification of Bacterial Populations

Wheat cv. Yangmai 158 was planted in a greenhouse with a 12 h photoperiod and cultivated at 28^∘^C with 75% RH. The bacterial suspentions were prepared by inoculating cells into 5 ml NA broth containing the appropriate antibiotics. Bacterial cells were grown for 12 h, harvested, washed and suspended in 10 mM MgCl_2_ to OD_600_ = 0.2 (1.6 × 10^8^CFU/ml). To access the *tal* contribution, wheat plants (3 weeks old) were spray-inoculated with the wild-type (WT), *tal* mutants (Δ*tal1*, Δ*tal2*, and *tal-*free) and *tal* complemented mutants (PHZW-*tal1* and PHZW-*tal2*) with a hand atomizer, which simulates natural entry of the pathogen into stomates, hydathodes and lenticels. Symptoms were compared at 11 days post-inoculation (dpi). For the T3SS virulence assays, the WT, Δ*hrcC* mutant, and Δ*hrcC*/*hrcC* were spray-inoculated into young leaves of 3 weeks old wheat plants and needless syringe infiltrated into 3 weeks old *N. benthamiana* plants; symptoms were evaluated at 11 dpi (wheat) and 24 hpi (*N. benthamiana*).

For quantification of bacterial growth, three replicate leaf samples (2 × 0.5 cm) were excised from five plants and surface-sterilized in 70% ethanol and double-distilled water (ddw). Samples were macerated in 1 ml ddw and incubated at 4^∘^C for 1 h; serial dilutions were then plated for enumeration of bacteria. The experiment was repeated at least three times, and the Student’s *t*-test was used to determine significant difference.

## Results

### *X. translucens* pv. *cerealis* NXtc01 Genome Sequence

The NXtc01 genome is encoded by a 4,622,298 bp chromosome that lacks autonomous plasmid DNA (Figure 1). The genome, as annotated by the NCBI Prokaryotic Genome Annotation Pipeline (PGAP), contains overall G + C content is 67.23%, and 4,004 coding DNA sequences (CDS) with an average length of 1,011 bp. NXtc01 has two rRNA operons, 54 tRNA genes and other non-coding RNA genes, which are characteristics similar to other *Xanthomonas* genomes (Table 1). NXtc01 genome consists of 0.13% repetitive regions of 6,269 bp. A total of 43 simple repeat sequences and one satellite sequence were predicted via Repbase. In genome 80 complete and 56 partial insertion sequence (IS) elements were predicted using the ISfinder database (Table 1). To access the completeness and contamination of this genome CheckM was used, which is 99.86 and 0.05%. These results revealed the high quality assembled genome.

**TABLE 1:** Comparison of *X. translucens* pv. *cerealis* genomes from strains NXtc01 and CFBP 2541, which were isolated from *Triticum aestivum* in Xinjiang, China and *Bromus inermis* in the United States, respectively.

**Contents**	**NXtc01**	**CFBP 2541**
Genome size (bp)	4,622,298	4,518,140
GC%	67.23	67.34
Number of CDS	4,004	3,569
TAL effector genes	2	2
Non-TALE T3E genes	35	33
rRNA operons	2	2
tRNA genes	54	53
Insertion sequence elements (complete/partial)	80/56	88/58
CRISPR array	1	3

**FIGURE 2:**
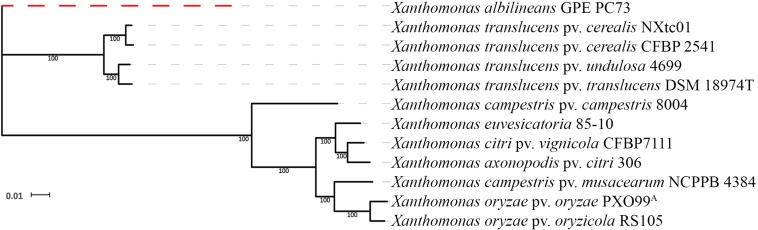
Phylogenetic relationships based on single copy genes. The tree was constructed from a concatenated alignment of 1,270 single copy genes and shows the relationship of NXtc01 to the following *Xanthomonas* spp. (accession numbers in parenthesis): *Xtc* CFBP 2541 (PRJNA268946); *X. translucens* pv. *undulosa* 4699 (PRJNA248137); *X. translucens* pv. *translucens* DSM 18974T (PRJEB647); *X. campestris* pv. *campestris* 8004 (PRJNA15); *X. euvesicatoria* 85-10 (PRJNA341901); *X. citri* pv. *vignicola* CFBP7111 (PRJNA390891); *X. axonopodis* pv. *citri* 306 (PRJNA297); *X. campestris* pv. *musacearum* NCPPB 4384 (PRJNA73881); *X. oryzae* pv. *oryzae* PXO99^A^ (PRJNA131967); and *X. oryzae* pv. *oryzicola* RS105 (PRJNA280380). *X. albilineans* GPE PC73 (PRJEA16687) was used as an outgroup.

Genome-wide, single-copy gene comparisons were made between NXtc01, the draft genome of *Xtc* CFBP 2541 (accession no. PRJNA268946) and other *Xanthomonas* genome sequences [*X. albilineans* GPE PC73, PRJEA16687 (Pieretti et al., 2009); *X. translucens* pv. *undulosa* 4699, PRJNA248137 (Peng et al., 2016); *X. translucens* pv. *translucens* DSM 18974T, PRJEB647 (Jaenicke et al., 2016); *X. campestris* pv. *campestris* 8004, PRJNA15 (Qian et al., 2005); *X. euvesicatoria* 85-10 (previously known as *X. campestris* pv. *vesicatoria*) PRJNA341901 (Thieme et al., 2005); *X. citri* pv. *vignicola* CFBP7111, PRJNA390891 (Ruh et al., 2017a); *X. axonopodis* pv. *citri* 306, PRJNA297 (da Silva et al., 2002); *X. campestris* pv. *musacearum* NCPPB 4384, PRJNA73881 (Wasukira et al., 2012); *X. oryzae* pv. *oryzae* PXO99^A^, PRJNA131967 (Salzberg et al., 2008); *X. oryzae* pv. *oryzicola* RS105, PRJNA280380 (Wilkins et al., 2015)]. This analysis revealed that NXtc01 groups with *Xtc*, *Xtu* and *Xtt* and resides on the same branch as *Xtc* CFBP 2541 (Figure 2). Thus, *Xtc* NXtc01 and CFBP 2541 are closely related, even though they were isolated from different continents.

### Comparative Analysis of NXtc01 and CFBP 2541 Genomes

**FIGURE 3:**
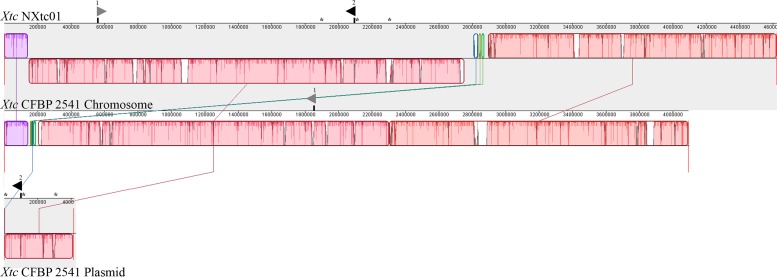
Organization of the whole genome and *tal* genes of *Xtc* strains NXtc01 and CFBP 2541. Genomes starting from *gyrB* gene were aligned using progressive MAUVE with default parameters (http://darlinglab.org/mauve). Horizontal axes show sequence coordinates in base pairs. The colored, locally collinear blocks (LCB) shows conserved and highly similar genomic regions; white areas inside colored regions indicates sequence elements specific to one genome that were not aligned. Height of similarity profile is present inside each block. The colored lines that connect LCBs represent translocations of homologous regions. Blocks above or below the horizontal bar indicate regions that aligned in the forward or reverse orientation, respectively (same-colored LCBs on opposite side of the center line indicates an inversion). Asterisks indicates IS elements that bordered *tal2*. The two *tal* genes are represented as triangles above the sequence coordinates, pointing toward transcriptional orientation on chromosomal or plasmid DNA. Black filled triangles indicate *tal*2 on chromosome in NXtc01 and on plasmid in CFBP 2541, encode identical RVDs. Gray filled triangles indicate *tal1* (see Table 2 for RVD sequences).

A comparison of the NXtc01 genome with the draft genome of CFBP 2541 is illustrated in Figure 3 starting from same gene, *gyrB*. The structure of both genomes is quite similar but with a single giant inversion that is bordered on one side with putative IS elements and/or integrases. The NXtc01 genome is 104 kb larger than CFBP 2541, however, this could be due to incomplete sequencing in the draft genome of CFBP 2541. Both strains harbor two *tal* genes that are identical in size but located in different genomic positions. CFBP 2541 contains a plasmid-borne TALE gene, named *tal2* (Pesce et al., 2015), whereas the gene is chromosomally encoded in NXtc01, possibly due to horizontal transfer (Figure 3). IS elements are frequently found in both genomes (Table 1); implicating possible involvement in the gain or loss of some regions. Likely, IS elements found at the CFBP 2541 plasmid and NXtc01 chromosome regions containing *tal2* (Figure 3) indicating the introgression that might be transposon mediated. The genomes of *Xtc* NXtc01 and CFBP 2541 also contained one and three CRISPR array^2^, respectively (Table 1).

**FIGURE 4:**
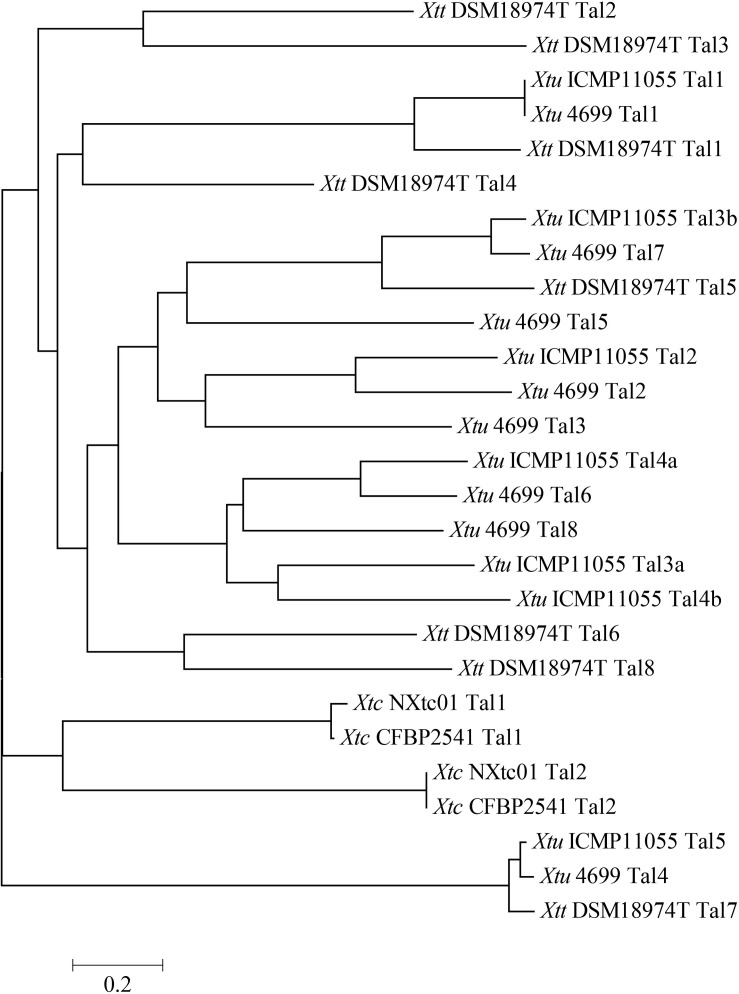
Phylogenetic tree based on TALEs repeat arrangement alignments. DisTAL was used to build tree for a set of 27 TAL effector sequences from 5 different *X. translucens* strains. TALEs were classified into 17 groups showing the relationship of *Xtc* NXtc01 to several other *X. translucens* strains published previously (Pesce et al., 2015; Jaenicke et al., 2016; Peng et al., 2016; Falahi Charkhabi et al., 2017). Scale is shown below the tree.

TALE phylogenetic tree of *X. translucens* strains were created by aligning TALE-CRR using DisTAL. All 27 TALEs (*Xtc* NXtc01 = 2, *Xtc* CFBP 2541 = 2, *Xtu* ICMP11055 = 7, *Xtu* 4699 = 8 and *Xtt* DSM18974T = 8) were classified into 17 groups. Both Tal1 and Tal2 of *Xtc* stains NXtc01 and CFBP 2541 grouped separately from pv. *translucens* and pv. *undulosa* TALEs (Figure 4). Together, two of these groups contained nearly identical TALEs from all *X. translucens* strains, while all others were exclusive to *Xtu* or *Xtt* strains (Figure 4).

In Nxtc01, Tal1, and Tal2 contained 11.5 and 15.5 RVDs, respectively (Table 2). Tal1 in NXtc01 and CFBP 2541 contained 11.5 RVDs that were nearly identical except for variation in the RVD of the third repeat (Table 2). Tal1 in NXtc01 and CFBP 2541 RVDs did not show relatedness to TALEs annotated in *Xtu* 4699, *Xtt* DSM 18974T, or *Xtu* ICMP11055. Tal2 consisted of 15.5 RVDs that were identical in *Xtc* NXtc01 and CFBP 2541; Tal2 was also closely related to Tal4, Tal5, and Tal7 in *Xtu* 4699, *Xtu* ICMP11055, and *Xtt* DSM 18974T, respectively (Table 2).

**TABLE 2:** Comparison of RVDs in Tal1 and Tal2 with RVDs in other *X. translucens* strains and pathovars.

**TALEs**	**1**	**2**	**3**	**4**	**5**	**6**	**7**	**8**	**9**	**10**	**11**	**12**	**13**	**14**	**15**	**16**
*Xtc* NXtc01 Tal1	NS	KI	NN	HD	NK	GI	HD	NK	HD	NN	**HD**	NK				
*Xtc* CFBP2541 Tal1	NS	KI	NI	HD	NK	GI	HD	NK	HD	NN	**HD**	NK				
*Xtc* NXtc01 Tal2	NN	NN	KI	NN	HD	NG	HD	NG	NG	NK	HD	HD	**NN**	QD	**NG**	QD
*Xtc* CFBP2541 Tal2	NN	NN	KI	NN	HD	NG	HD	NG	NG	NK	HD	HD	**NN**	QD	**NG**	QD
*Xtu* 4699 Tal4	NH	NN	HD	NN	HD	NH	HD	YK	NG	NH	Y^∗^	HD	NN	NI	NG	QD
*Xtt* DSM18974T Tal7	NH	NN	HD	NN	HD	NH	HD	YK	NG	NH	Y^∗^	HD	NN	NI	NG	QD
*Xtu* ICMP11055 Tal5	NH	NN	HD	NN	HD	NH	HD	YK	NG	NH	Y^∗^	HD	NN	NI	NG	QD

**The sequences used for comparison to Xtc NXtc01 TALEs were published previously in GenBank under the accession numbers indicated in parentheses: Xtc CFBP 2541 (PRJNA268946); Xtu 6499 (PRJNA248137); Xtt DSM 18974T (PRJEB647); and Xtu ICMP11055 (PRJNA264445). Tal1 and Tal2 in NXtc01 contain 11.5 and 15.5 RVDs, respectively. The NS RVD in Tal1, which is also conserved in X. oryzae TALEs, is shown in green font. RVDs that differentiate Xtc from other X. translucens pathovars are indicated in yellow-highlighted font. The underlined RVD (NI, CFBP 2541 Tal1) is not found in NXtc01. RVDs having a repeat of 34 aa instead of 35 aa are shown in blue font. An asterisk indicates the missing aa of RVD. This analysis was done using AnnoTALE version 1.2.**

In addition to Tal1 and Tal2, the *Xtc* NXtc01 and CFBP 2541 genomes encode 35 and 33 non-TALE T3Es, respectively. Many of these non-TALE genes are Xop-encoding genes that are also conserved in *Xtu* ICMP11055 (Falahi Charkhabi et al., 2017), *Xtt* DSM 18974T (Jaenicke et al., 2016), *X. campestris* pv. *campestris* 8004 (Qian et al., 2005), *Xoo* PX099^A^ (Salzberg et al., 2008), and *Xoc* BLS256 (Bogdanove et al., 2011; Supplementary Table S3).

### Type III Secretion System (T3SS) in *X. translucens* pv. *cereali*s

The organization of the T3SS in *Xtc* NXtc01 was identical to *Xtc* CFBP 2541, *Xtu* ICMP11055 and *Xtt* DSM 18974T (Supplementary Figure S2). Interestingly, we identify putatively encoding an *hrp* gene cluster covering 20,486 bp region (from *hpaD* to *hrcC*) in *Xtc* NXtc01. This region consisted of eight *hrp*, 11 *hrc* and four *hpa* genes (Supplementary Figure S2). The 23 genes that encode this cluster are conserved among the three *X. translucens* pathovars (Supplementary Figure S2), but have less similarity based on aa identity and genetic organization at the species level. The two *hrp* regulatory genes, *hrpG* and *hrpX*, flank the *hrp* cluster of NXtc01 (Supplementary Figure S2), and this organization is conserved in other *X. translucens* pathovars (Supplementary Figure S2; Falahi Charkhabi et al., 2017; Pesce et al., 2017).

**FIGURE 5:**
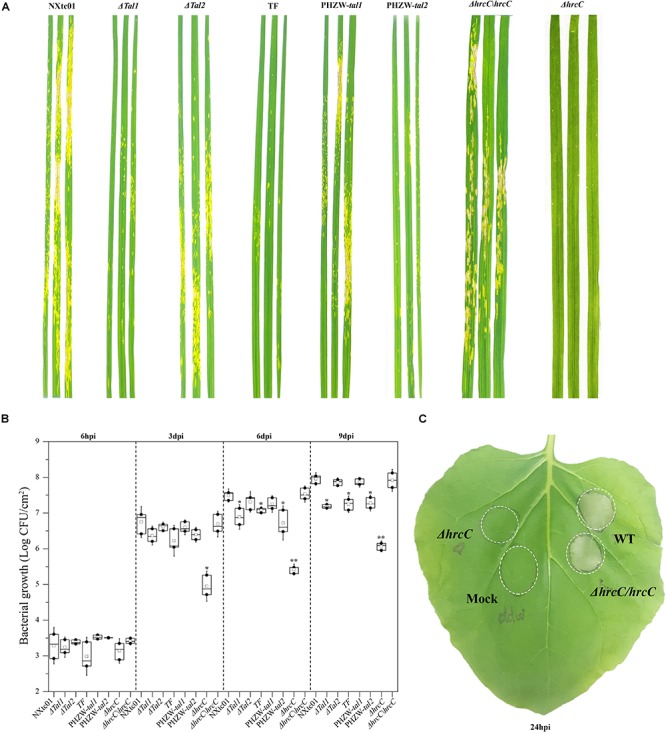
Aggressiveness and bacterial growth of wild type *Xtc* NXtc01, deletion mutants and complemented strains. NXtc01 (WT), Δ*tal1*, Δ*tal2*, *tal*-free (TF), Δ*hrcC*, PHZW-*tal1*, PHZW-*tal2*, and Δ*hrcC/hrcC* were sprayed-inoculated (OD_600_ = 0.2) onto 3 weeks old wheat plants. **(A)** Symptoms were compared and photographed at 11 dpi. **(B)** Bacterial populations were quantified at 6 hpi and 3, 6, and 9 dpi. The box plot displays replicates (*n* = 3) and mean value (middle box); error bars show mean ± SD. ^∗^*p* < 0.05, ^∗∗^*p* < 0.01 (Student’s *t*-test); indicates a significant difference compared to WT and Δ*hrcC/hrcC*. **(C)** The inocula (OD_600_ = 0.2) of WT, Δ*hrcC*, Δ*hrcC/hrcC*, and mock (ddw) were infiltrated into three to four leaves of 20 days old *N. benthamiana* plants using a needless syringe, and infiltrated areas were marked. Symptoms were observed and compared at 24 hpi. **(A–C)** Experiments were repeated at least three times with similar results. WT, *Xtc* NXtc01 (wild-type); Δ*tal1*, NXtc01 *tal1* deletion mutant; Δ*tal2*, NXtc01 *tal2* deletion mutant; TF, NXtc01 *tal*-free mutant; Δ*hrcC*, NXtc01 *hrcC* deletion mutant; PHZW-*tal1*, NXtc01 TF with *tal1 in trans*; PHZW-*tal2*, NXtc01 TF with *tal2 in trans;* Δ*hrcC/hrcC*, NXtc01 Δ*hrcC* containing *hrcC in trans*.

We deleted the *hrcC* gene, which encodes an outer ring protein of the T3SS (Li et al., 2017), and evaluated the phenotypes of the Δ*hrcC* mutant on wheat and the non-host *N. benthamiana*. In contrast to the virulent NXtc01 strain, the Δ*hrcC* mutant was non-pathogenic on wheat cv. Yangmai 158 and did not elicit an HR on *N. benthamiana* (Figures 5A,C). For complementation analysis, a recombinant plasmid containing the entire coding region of *hrcC* (1,848 bp) was constructed and designated pH1-Flag(-)*hrcC* (Supplementary Table S1). This plasmid was introduced into Δ*hrcC*, resulting in strain Δ*hrcC*/*hrcC*; production of HrcC *in trans* was confirmed by western blot analysis (Supplementary Figure S1C). The pathogenicity of Δ*hrcC*, Δ*hrcC*/*hrcC* and wild-type NXtc01 were compared by assessing disease symptoms in wheat leaves at 11 dpi, and an HR on *N. benthamiana* at 24 hpi. Both the wild-type and the complementing strain Δ*hrcC*/*hrcC* produced similar symptoms on wheat and the HR on *N. benthamiana*, indicating that the *hrp* phenotype was restored to Δ*hrcC* when the *hrcC* gene was supplied *in trans* (Figures 5A,C).

In addition, we also test the bacterial proliferation of Δ*hrcC* and Δ*hrcC*/*hrcC in planta*. The bacterial multiplication in mutant Δ*hrcC* was reduced as compared to virulent NXtc01 strain. While Δ*hrcC*/*hrcC* have restored bacterial growth similar like wild type strain NXtc01 (Figure 5B).

### Non-TALEs of NXtc01

The NXtc01 genome contained 35 non-TALEs; 10 are conserved T3Es, including AvrBs2, XopF, XopK, XopL, XopN, XopP, XopQ, XopR (frameshift mutation), XopX, and XopZ (Supplementary Table S3). *X. translucens* pathovars contained multiple copies of AvrBs2 (*n* = 2), XopF (*n* = 2), XopL (*n* = 2–4), XopP (*n* = 2–4) and XopX (*n* = 3) (Supplementary Table S3). Other T3Es conserved in *X. translucens* pathovars include XopAA, XopAD, XopAF1, XopAM, XopAP, XopB, XopC2, XopG1, XopV, and XopY. The T3Es varied among *Xanthomonas* species, pathovars and strains. For example, AvrBs1 is present in *Xtc* CFBP 2541 and *Xcc* 8004 but was not present in NXtc01 (Supplementary Table S3); whereas AvrXccA1, XopAZ, and XopJ were detected in NXtc01 but not in CFBP 2541. AvrXccA1 and XopAZ were present in NXtc01 but not the other *X. translucens* pathovars, and XopJ was only present in NXtc01 and DSM 18974T.

### TALEs of NXtc01

The number (*n* = 2) of TALEs in *Xtc* Nxtc01 were confirmed by Southern blot analysis (Supplementary Figure S1B). Furthermore, we also sequenced the *tal* genes after cloning hybridizing *Bam*HI fragments; the corresponding sequences were identical to the *tal* genes in the genome. The sizes of TALE genes are; *tal1* 2,898 bp and *tal2* 3,342 bp but *tal2* N-terminus lack classically conserved *Bam*HI site (Supplementary Figure S1B). The two TALEs encode 11.5 and 15.5 RVDs, respectively. Some unusual RVDs found in Tal1 and/or Tal2 include GI and KI, which help differentiate *Xtc* from other *Xanthomonas* spp. (Table 2), and QD, which is occurring once in TALEs of *X. oryzae* pv. *oryzicola* CFBP 7331 and CFBP 7341 (Wilkins et al., 2015). Tal1 contains the NS RVD, which is common in *X. oryzae*, and Tal2 contains QD at the distal end, which is conserved in other *X. translucens* pathovars (Table 2). The Tal1 and Tal2 RVDs in NXtc01 are identical to those in *Xtc* CFBP 2541 strain except for the third RVD in Tal1 (Table 2).

### Tal1_NXtc__01_ Contribution to Virulence

To examine whether the NXtc01 TALEs contribute to virulence, *tal* deletion mutants were generated by homologous recombination using pKMSTal1 and pKMSTal2. Putative mutants were initially screened via colony PCR using pKMSTal1 and pKMSTal2 as positive controls (Supplementary Figure S1A). The screened mutants were further verified by Southern blot hybridization with a DIG-labeled *Sph*I fragment of *tal1*. The deletion mutants were designated Δ*tal1*,Δ*tal2* and TF (*tal*-free mutant) (Supplementary Figure S1B). Suspensions of the wild-type NXtc01, Δ*tal1*, Δ*tal2*, and the *tal*-free mutant were sprayed on leaves of wheat cv. Yangmai 158, and symptoms were evaluated at 11 dpi. The Δ*tal1* and *tal-*free mutants showed a significant reduction in disease symptoms as compared with the wild-type strain (Figure 5A), and the population growth of these two mutants was significantly lower than the wild-type (Figure 5B). In contrast, the Δ*tal2* mutant was not impaired in symptom development, and its growth *in planta* was not significantly different from the wild-type NXtc01 (Figures 5A,B).

To further investigate the contribution of *tal* genes in virulence, we expressed individual *tal* genes in the *tal*-free mutant. Recombinant plasmids containing *tal1* and *tal2* were constructed and designated pHZW-*tal1* and pHZW-*tal2* (Supplementary Table S1), respectively, and introduced into the NXtc01 *tal-*free strain. The expression of individual *tal* genes in the *tal*-free mutant was confirmed by immunoblotting (Supplementary Figure S1C). The introduction of *tal1*, but not *tal2*, restored full virulence to the *tal*-free mutant (Figure 5A) and its multiplication *in planta* (Figure 5B); indicating that *tal1* contributes to NXtc01 virulence in wheat.

## Discussion

In this study, we sequenced the genome of *Xtc* NXtc01, a virulent strain isolated from wheat grown in Xinjiang, China. The NXtc01 genome was compared with the draft genome of *Xtc* CFBP 2541 and other strains of *X. translucens*, *X. campestris*, and *X. oryzae*. The *Xtc* NXtc01 and CFBP 2541 genomes are slightly different in sequence and size, and show one giant rearrangement relative to each other. Interestingly, *tal2* is chromosomally-encoded in NXtc01 but plasmid-borne in CFBP 2541, which is likely due to transposon mediated horizontal gene transfer (Siguier et al., 2006; Ruh et al., 2017b; Chen et al., 2018). Comparative analysis based on single-copy genes revealed homogeneity among *X. translucens* strains; in other words, *X. translucens* are divided into two subgroups apart from *X. campestris* and *X. oryzae*. *Xtc* grouped together uniquely, beside a group of *Xtu* and *Xtt*. On the basis of genetic similarity, this result suggests that *Xtc* is a robust biological entity with a common genetic basis for host specificity apart from their geographical distribution.

In many phytopathogenic bacteria, the T3SS is critical for virulence and modulates the delivery of effector proteins into plant cells. The T3SS of group 1 and group 2 *Xanthomonas* are very different; for example, *X. albilineans* belongs to group 1 and has a T3SS different from that encoded by the *hrp* gene cluster. *Xanthomonas* spp. in group 2 encode a typical Hrp T3SS (Marguerettaz et al., 2011), which is comprised of seven *hrp* operons, 11 conserved *hrc* (*hrp-*conserved) genes and other *hpa* (*hrp-*associated) genes (Tampakaki et al., 2010; Li et al., 2017). The products of the nine *hrc* genes are structural components of the T3SS for secretion of T3Es into plants (Li et al., 2017). A comparison of genes encoding the T3SS in seven different *Xanthomonas* strains revealed that the organization of the cluster in NXtc01 and other *X. translucens* strains is conserved (Supplementary Figure S2). Notably, the organization of regulatory genes *hrpG* and *hrpX* in NXtc01 is different from the position of *hrpG/X* in *Xcc* 8004, *Xoo* PXO99^A^, and *Xoc* BLS256 (Supplementary Figure S2). Interestingly, a genetic organization similar to NXtc01 has been reported for the T3SS in *R. solanacearum* for *hrpB* (homologous to *hrpX*) and *hrpG* (Salanoubat et al., 2002; Pesce et al., 2017).

In addition, we also demonstrated that a mutant of the conserved *hrp* gene *hrcC* of wheat pathogen *Xtc* NXtc01 resulted completely loss of symptoms and hardly colonize the leaf upon inoculation (Figures 5A,B), similar as barley pathogens *Xt* pv. *hordei* and pv. *translucens hrcT* mutant and wheat pathogen *Xtu* XT4699 *hrcC* mutant, respectively, a typical phenotype like group 2 *Xanthomonas* (Peng et al., 2016; Pesce et al., 2017). In contrast grass pathogen *Xtg*29 *hrcR*, *hrcE*, and *hrpG* mutants can’t eliminate the symptoms completely and colonization is also unaffected (Wichmann et al., 2013). The *hrc* gene products are structural components of the T3SS, which functions as a conduit for secretion of T3Es into plants (Kim et al., 2003; Li et al., 2017). Possibly the T3SS import more T3Es into small grain cereals than the grasses for colonization and symptoms development. Therefore, it will be worthwhile to compare the *hrcC* or *hrcT* mutants in all other *X. translucens* pathovars.

We extended our analysis of mutant *hrcC* in non-host *N. benthamiana*. Infiltration of *Xtc* NXtc01 T3SS-deficient mutant, a Δ*hrcC* does not show any visible HR. while the complementation showed non-host HR, similar like wild type (Figure 5C). Thus non-host HR largely depends on translocation of T3Es, likely XopQ (Adlung et al., 2016).

This study also reveals a repertoire of non-TALEs in NXtc01 that have been identified in other *Xanthomonas* spp. *Xtc* NXtc01 and *X. translucens* strains contain two copies of *AvrBs2* (Supplementary Table S3), which contains a putative glycerophosphoryl-diester phosphodiesterase domain thought to be involved in plant host signaling and osmotic adaptation (Swords et al., 1996; Li et al., 2015). *X. translucens* strains also contain a single copy of XopK and two copies of XopF (Supplementary Table S3); these effectors presumably suppress *Xoo-*mediated PAMP-triggered immunity in rice (Mondal et al., 2016; Qin et al., 2018). *Xtc* NXtc01 and CFBP 2541 both contain copies of XopL and XopN, which were essential for *X. axonopodis* pv. *phaseoli* virulence (Kumar et al., 2016; Soni and Mondal, 2018). XopL encodes an E3 ubiquitin ligase that interacts with plant-specific E2 enzymes and ubiquitinates unidentified target proteins in *X. euvesicatoria* 85-10 (Singer et al., 2013). Several other non-TALE core T3Es of *Xanthomonas* that play important roles, likely XopR in *Xoo* strain 13571 is required for full virulence but the mechanism is yet not understood (Zhao et al., 2013). Another core effector, the XopZ that potentially interferes the bacterial proliferation and host innate immunity in *Xoo* PXO99^A^ (Song and Yang, 2010). XopQ of *Xoo* strain BXO43 suppressing the rice immune responses by interacting Gf14f and Gf14g, the two rice 14-3-3 proteins (Deb et al., 2019). XopP hijacked the proteasomal pathway by interacting with E3 ubiquitin ligase PUB44 in *Xoo* (Ishikawa et al., 2014). Less conserved T3Es, such as XopB, were shown to be important in the multiplication of *X. euvesicatoria* 85-10 *in planta* and inhibited the immune responses trigged by other T3Es (Schulze et al., 2012). Other T3Es, notably XopY, XopAA, and XopAP (Yamaguchi et al., 2013a, b; Teper et al., 2016), also provide valuable avenues of research for probing the roles of these effectors in NXtc01. Furthermore, the conserved sets of *X. translucens* non-TALEs may ultimately help us identify pyramided *R* genes for BLS resistance.

Plant diseases are driven by molecular interactions between pathogen effectors and host defense genes, and there is evolutionary pressure for pathogens to develop new virulence strategies to counteract host defense mechanisms (Jones and Dangl, 2006). The pathogenesis of *Xanthomonas* spp. largely depends on T3Es that are translocated into plant cells by the T3SS (Gürlebeck et al., 2006). In order to understand how *X. translucens* TALome (TALEs per genome) differ from each other within and between strains, different tools were applied (Pérez-Quintero et al., 2015; Grau et al., 2016). *X. translucens* encodes very diverse TAL effectors that were classified exclusively into 17 groups. TALE phylogenetic tree showed that all *X. translucens* pathovars form distinct groups, despite the overall genomic similarities (Figure 4).

In this study, we investigated the role of two TALEs, *tal1* and *tal2*, in the virulence of *Xtc* NXtc01; *tal1*, but not *tal2*, was required for a full level of virulence. Although the TALE repertoire of *X. translucens* pathovars is somewhat variable, *tal2*_NXtc__01_ was highly similar to *tal2* in CFBP 2541, *tal4* in *Xtu* 4699, *tal5* in *Xtu* ICMP11055, and *tal7* in *Xtt* DSM 18974T with respect to the number and sequence of RVDs and repeat length. Interestingly, the TALEs in *Xtc* NXtc01 share some features (e.g., repeat length, RVD composition) with the RipTALs of *R. solanacearum*, which may be the result of convergent evolution and/or recombination events (Schandry et al., 2016).

Numerous reports show that *tal* genes contribute to virulence in *Xtu, Xoc, Xoo, X. euvesicatoria, X. campestris* pv. *malvacearum*, *X. citri* subspecies *citri*, *X. axonopodis* pv. *vesicatoria*, and *X. axonopodis* pv. *manihotis* (Yang, 1994; Wichmann and Bergelson, 2004; Antony et al., 2010; Cernadas et al., 2014; Cohn et al., 2014; Hu et al., 2014; Cox et al., 2017; Falahi Charkhabi et al., 2017). The NXtc01 genome provides an excellent platform for future comparative genomic studies aimed at understanding *Xtc* pathogenicity in wheat. Deletion mutagenesis of NXtc01 and complementation analysis with the *tal*-free strain clearly demonstrated that *tal1*_NXtc__01_ contributes to virulence on wheat. The deletion mutants and genetic constructs described in this study provide valuable new tools to identify the wheat gene(s) that is potentially activated by Tal1_NXtc__01_.

## Data Availability

The datasets generated for this study can be found in NCBI, PRJNA528834.

## Author Contributions

SS and GC designed the study, with assistance from FH, WM, XX, and ZX. SS conducted the experiments, with assistance from FH, XX, WM, and LZ. SW and BZ did phylogenetic analysis and also helped in other computational analysis. SS and GC prepared the manuscript. All authors read and approved the final version of the manuscript.

## Conflict of Interest Statement

The authors declare that the research was conducted in the absence of any commercial or financial relationships that could be construed as a potential conflict of interest.

## Footnotes

1 https://orthomcl.org/orthomcl/

2 https://crispr.i2bc.paris-saclay.fr/
