# Supplementary material for: Tal1NXtc01 in Xanthomonas translucens pv. cerealis Contributes to Virulence in Bacterial Leaf Streak of Wheat
Source: Front Microbiol. 2019 Sep 4;10:2040. doi: 10.3389/fmicb.2019.02040 (PMC6737349; doi:10.3389/fmicb.2019.02040)
Supplement: Supplementary file 1 [file Data_Sheet_1.docx]

Supplementary Material

Tal1_NXtc01_ in *Xanthomonas translucens* pv. *cerealis* Contributes to Virulence in Bacterial Leaf Streak of Wheat

Syed Mashab Ali Shah^1^, Fazal Haq^1^, Wenxiu Ma^1^, Xiameng Xu^1^, Sai Wang^1^, Zhengyin Xu^1^, Lifang Zou^1,2^, Bo Zhu^1,2^ and Gongyou Chen^1,2^*

^1^School of Agriculture and Biology, Shanghai Jiao Tong University/Key Laboratory of Urban Agriculture by the Ministry of Agriculture of China, Shanghai, 200240, China

^2^State Key Laboratory of Microbial Metabolism, School of Life Science and Biotechnology, Shanghai Jiao Tong University, Shanghai, 200240, China

*** Correspondence:** Gongyou Chen

[gyouchen@sjtu.edu.cn](mailto:gyouchen@sjtu.edu.cn)

**Running title:** Tal1_NXtc01_ contributes to virulence in *X. translucens* pv. *cerealis*

Supplementary Tables

Table S1. Bacterial strains and plasmids used in this study.

| Strains and plasmids | | Relevant characteristics | Source |
| --- | --- | --- | --- |
| Strains | | | |
| *Xanthomonas translucens* pv. *cerealis* | | | |
| NXtc01 | Wild-type, isolated from *Triticum aestivum* in Xinjiang province, China, 2016 | | This study |
| Δ*tal1* | *tal1* knockout mutant of NXtc01 | | This study |
| Δ*tal2* | *tal2* knockout mutant of NXtc01 | | This study |
| TF | *tal* free mutant of NXtc01 | | This study |
| PHZW-*tal1* | Ap^r^ Sp^r^, TF containing *tal1* *in trans* | | This study |
| PHZW-*tal2* | Ap^r^ Sp^r^, TF containing *tal2* *in trans* | | This study |
| Δ*hrcC* | *hrcC* knockout mutant of NXtc01 | | This study |
| Δ*hrcC*/*hrcC* | Sp^r^, *ΔhrcC* containing *hrcC* gene *in trans* | | This study |
| *Escherichia coli* | | |  |
| DH5α | F^-^ *endA1*, *thi-1*, *recA1*, Φ80*lacZ*, ΔM15 | | This lab |
| Plasmids |  | |  |
| pKMS1 | Km^r^, pUC18 polylinker, *mob*, *oriV*, *sacB* | | (Zou et al., 2011) |
| pB | Ap^r^, pBluescript II phagemid, pUC derivative | | This lab |
| pHM1 | Sp^r^, cosmid vector, *cos*, *parA*, *IncW* | | (Hopkins et al., 1992) |
| pZW | Ap^r^, pBluescript II with FLAG-tag N- (831-bp) and C- (363-bp) terminus of *avrXa10.* Lacks the *Sph*I fragment containing the CRR; for expression of TAL effector genes | | (Yang et al., 2000) |
| pB-*tal1* | Ap^r^, pB containing *tal1* at *Bam*HI site | | This study |
| pB-*tal2* | Ap^r^, pB containing *tal2* at *Bam*HI site | | This study |
| pKMSTal1 | Km^r^, pKMS1 containing sequences at the 5’ and 3’ end of *tal1*; used for generating *tal1* deletion mutant | | This study |
| pKMSTal2 | Km^r^, pKMS1 containing sequences at the 5’ and 3’ end of *tal2*; used for generating *tal2* deletion mutant | | This study |
| pKMShrcC | Km^r^, pKMS1 containing sequences at the 5’ and 3’ end of *hrcC*; used for generating *hrcC* deletion | | This study |
| pZW-*tal1* | Ap^r^, CRR of *tal1* from *Sph*I digested pB-*tal1* were ligated into *Sph*I-linearized, CIP-treated pZW vector | | This study |
| pZW-*tal2* | Ap^r^, CRR of *tal2* from *Sph*I digested pB-*tal2* were ligated into *Sph*I-linearized, CIP-treated pZW vector | | This study |
| pHZW-*tal1* | Ap^r^ Sp^r^, pZW with *tal1* in pHM1 at *Hin*dIII site | | This study |
| pHZW-*tal2* | Ap^r^ Sp^r^, pZW with *tal2* in pHM1 at *Hin*dIII site | | This study |
| pB-*hrcC* | Ap^r^, pBluescript II, contains *hrcC* | | This study |
| pH1-Flag(-) | Sp^r^, pHM1 containing MCS with FLAG-tag bacteriophage λ T0 and *E. coli* *rrnB* T1 terminators | | Yang Xiaofei, unpublished data |
| pH1-Flag(-)*hrcC* | Sp^r^, pH1**-**Flag(-); contains *hrcC* at *Sal*I/*Kpn*I sites | | This study |

**Note**: Ap^r^, ampicillin resistance; Km^r^, kanamycin resistance; Sp^r^, spectinomycin resistance.

Table S2. Primers used in this study.

| Primers | Sequence 5’to 3’ | Description |
| --- | --- | --- |
| T1Nt-F | AACTGCAGTGGATGGCTTGCCCGCTC | Amplifies 640-bp fragment upstream of *tal1*; used for deletion mutagenesis |
| T1Nt-R | GCTCTAGAGCCTGTGACCAACTGTAACG |  |
| T1Ct-F | GCTCTAGAGCAAGCATTTGATGAAGCG | Amplifies 425-bp fragment downstream of *tal1*; used for deletion mutagenesis |
| T1Ct-R | TCCCCCGGGGCGTTTTACACCCCAGCTACT |  |
| T2Ct-F | TCCCCCGGGAGGCAATAGCGTCATCA | Amplifies 698-bp fragment upstream of *tal2*; used for deletion mutagenesis |
| T2Ct-R | GCTCTAGACACAAGAACCCGTAACCT |  |
| T2Nt-F | GCTCTAGAATTATTGCTGACGATGGC | Amplifies 413-bp fragment downstream of *tal2;* used for deletion mutagenesis |
| T2Nt-R | AACTGCAGGGAGAAGATCAAACCGAAG |  |
| *hrcC*-up-F | TCCCCCGGGGTGGTCAGGCAGGTATCG | Amplifies 610-bp fragment upstream of *hrcC;* used for deletion mutagenesis |
| *hrcC*-up-R | CGCGGATCCCCAACGCCACTGTCAAA |  |
| *hrcC*-do-F | CGCGGATCCCGGGATTGTCCAAGGTG | Amplifies 694-bp fragment downstream of *hrcC;* used for deletion mutagenesis |
| *hrcC*-do-R | CTAGTCTAGAGGAAACTTCGCTGCCTC |  |
| M13-R | AGCGGATAACAATTTCACACAGG | Universal primers used for confirmation and sequencing |
| M13-F | CGCCAGGGTTTTCCCAGTCACGAC |  |
| *hrcC*-F1 | ACGCGTCGACATGAAACAGCAACGTTCGC | Amplifies 1217-bp fragment beginning at the start codon of *hrcC;* used for complementation in pH1-Flag(-) |
| *hrcC*-R1 | TCCAGATGGCGGTTGTG |  |
| *hrcC*-F2 | CGCACCGTTCAGATGGA | Amplifies 1260- bp fragment extending from the middle of *hrcC* and ending before stop codon; used for complementation in pH1-Flag(-) |
| *hrcC*-R2 | CGGGGTACCGGGCGAGACCACGTGG |  |
| TALN18S-F | AGAGGCGACACACGAAGACA | Amplifies 255-bp fragment, used to confirm of *Sph*I-ligated *tal* gene orientation in pZW |
| TalN-R | CCGCCACTATTGCTGACGAT |  |

**Note**: Underlined bases indicate restriction enzyme sites.

**Table S3|** Predicted type III effectors in *X. translucens* pv. *cerealis* NXtc01 based on comparison with other *Xanthomonas* spp. included *X. translucens* pv. *cerealis* CFBP 2541, *X. translucens* pv. *undulosa* ICMP11055, *X. translucens* pv. *translucens* DSM 18974T, *X. campestris* pv. *campestris* 8004, *X. oryzae* pv. *oryzae* PX099^A^ and *X. oryzae* pv. *oryzicola* BLS256.

| T3Es | NXtc01^a^ | CFBP2541^b^ | ICMP11055^c^ | DSM18974T^d^ | 8004^e^ | PXO99^Af^ | BLS256^g^ |
| --- | --- | --- | --- | --- | --- | --- | --- |
| AvrBs1 | ND | + | ND | ND | + | ND | ND |
| AvrBs2 | ++ | ++ | ++ | ++ | + | + | + |
| TALEs | 2 | 2 | 7 | 8 | ND | 19 | 28 |
| AvrXccA1 | + | ND | ND | ND | + | ND | ND |
| XopAA | + | + | + | + | ND | + | + |
| XopAD | + | + | + | + | ND | + | + |
| XopAF1 | + | + | + | ++ | ND | ND | + |
| XopAH | ND | ND | + | + | + | + | + |
| XopAK | ND | ND | + | + | ND | ND | + |
| XopAM | + | + | + | + | + | ND | ND |
| XopAP | + | + | + | F | ND | ND | + |
| XopAZ | + | ND | ND | ND | + | + | + |
| T3Es | **NXtc01^a^** | **CFBP2541^b^** | **ICMP11055^c^** | **DSM18974T^d^** | **8004^e^** | **PXO99^Af^** | **BLS256^g^** |
| XopB | + | + | + | + | ND | ND | ND |
| XopC2 | + | + | + | + | ND | + | + |
| XopE1 | + | + | + | ND | ND | ND | ND |
| XopE2 | + | + | ND | + | + | ND | ND |
| XopE3 | ND | ND | ND | + | ND | ND | ND |
| XopE5 | + | + | + | ND | ND | ND | ND |
| XopF | ++ | ++ | ++ | ++ | + | + | + |
| XopG | + | + | + | + | ND | + | ND |
| XopJ | + | ND | ND | + | + | ND | ND |
| XopK | + | + | + | + | + | + | + |
| XopL | ++++ | ++++ | ++ | ++ | + | + | + |
| XopN | + | + | + | + | + | + | + |
| XopP | +++ | +++ | ++ | +++ | + | + | ++ |
| XopQ | + | + | + | + | + | + | + |
| T3Es | **NXtc01^a^** | **CFBP2541^b^** | **ICMP11055^c^** | **DSM18974T^d^** | **8004^e^** | **PXO99^Af^** | **BLS256^g^** |
| XopR | F | F | + | + | + | + | + |
| XopV | + | + | + | + | ND | + | + |
| XopX | +++ | +++ | +++ | +++ | ++ | + | + |
| XopY | + | + | + | + | ND | + | + |
| XopZ | + | + | + | + | + | ++ | + |

Abbreviations: The number of plus symbols indicates the copy number of T3Es in a given strain. ND, not detected; F, frameshift mutation.

^a^ This study

^b^ (Pesce et al., 2015)

^c^ (Falahi Charkhabi et al., 2017)

^d^ (Jaenicke et al., 2016)

^e^ (Qian et al., 2005)

^f^ (Salzberg et al., 2008)

^g^ (Bogdanove et al., 2011)

**Table S4.** COG functional classifications and colors for Figure 1.

| **Single letter COG class** | **Functional description** | **Color in Figure 1** |
| --- | --- | --- |
| A | RNA processing and modification | Orange3 |
| B | Chromatin structure | Black |
| C | Energy production/conversion | RoyalBlue4 |
| D | Cell division/chromosome partitioning | AntiqueWhite1 |
| E | Amino acid transport/metabolism | DodgerBlue1 |
| F | Nucleotide transport/metabolism | SkyBlue3 |
| G | Carbohydrate transport/metabolism | Blue1 |
| H | Coenzyme transport/metabolism | LightBlue1 |
| I | Lipid transport/metabolism | Cyan3 |
| J | Translation/ribosomal structure | Gold1 |
| K | Transcription | DarkOrange1 |
| L | Replication/recombination/repair | DarkOrange3 |
| M | Cell wall/membrane/envelope biogenesis | PeachPuff3 |
| N | Cell motility | MediumPurple1 |
| O | Chaperones | PaleGreen1 |
| P | Inorganic ion metabolism | MediumPurple4 |
| Q | Secondary metabolite biosynthesis/transport/catabolism | Aquamarine4 |
| R | General function prediction | Gray90 |
| S | Function unknown | Gray70 |
| T | Signal transduction | Tomato1 |
| U | Intracellular trafficking/secretion | DeepPink |
| V | Defense mechanisms | Pink |
| W | Extracellular structures | Green |
| Y | Nuclear structure | Yellow |
| Z | Cytoskeleton | Red |
| None | No COG category | Gray50 |

Supplementary Figures


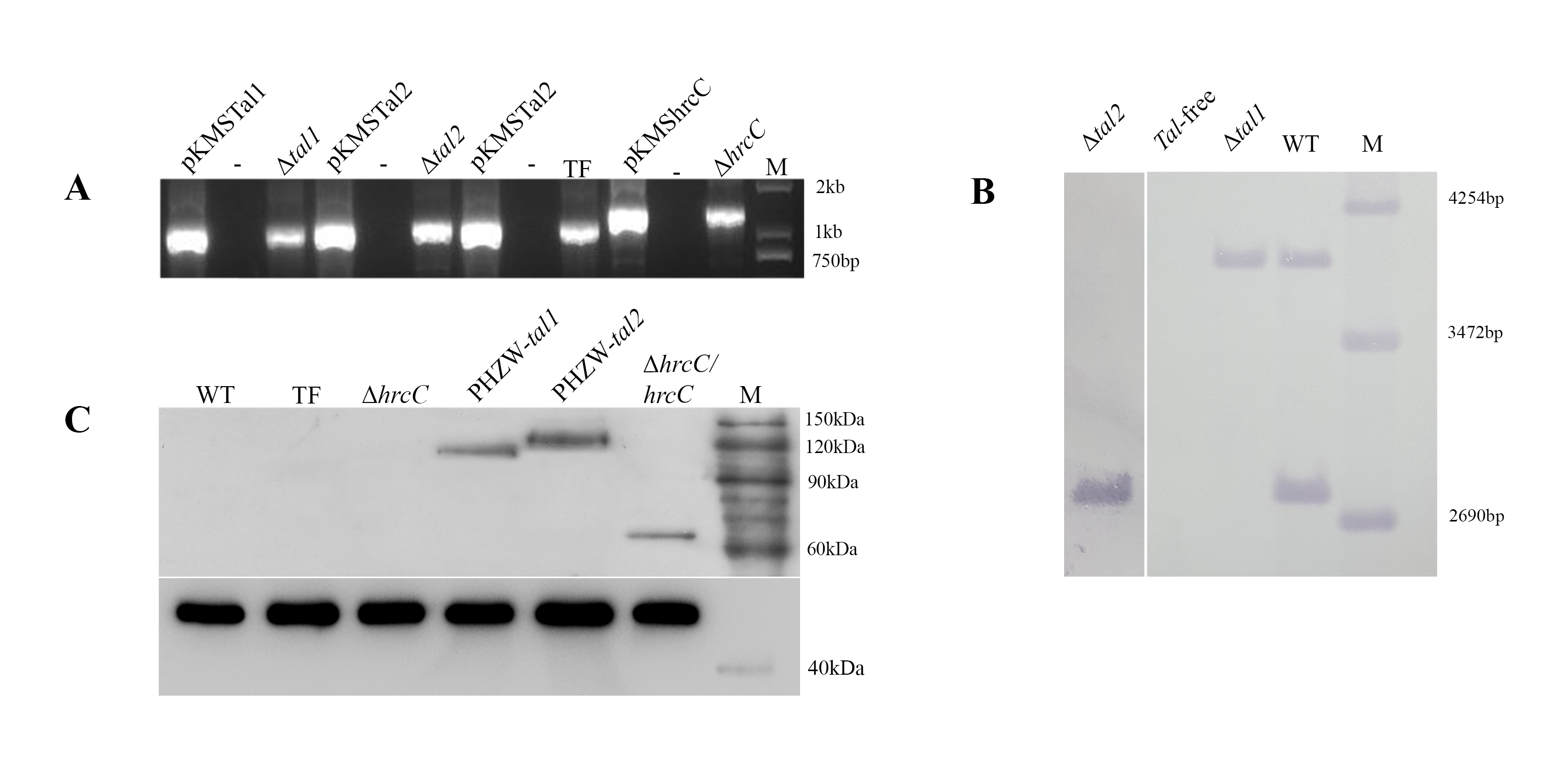


**Figure S1. Knockout and complementation analysis of *tal* and *hrcC* genes in NXtc01.** The *tal1*, *tal2*, *tal*-free and *hrcC* mutants were generated using suicide vector pKMS1. **(A)** Lanes show the constructs (pKMSTal1, pKMSTal2 and pKMShrcC) that were used as positive controls and mutants (Δ*tal1*, Δ*tal2*, TF and Δ*hrcC*) that were screened via PCR. Primers were as follows: T1Nt-F/T1Ct-R, amplifies a 1071-bp fragment; T2Ct-F/T2Nt-R, amplifies a 1124-bp fragment; and hrcC-up-F/hrcC-do-R, amplifies a 1317-bp fragment. **(B)** Southern blot of *Bam*HI-digested genomic DNA of WT and mutants, probed with a DIG-labeled *Sph*I fragment of the NXtc01 *tal1* gene. **(C)** Immunodetection of flag-tagged Tal1 (104.8 kDa) and Tal2 (119.3 kDa) in the *tal*-free mutant, and flag-tagged HrcC (66.8 kDa) in the Δ*hrcC* mutant. WT, *tal*-free and the Δ*hrcC* mutant were included as controls. Flag-tagged proteins were detected by autoradiography. An anti-E. coli RNA polymerase α antibody was used as loading control shown in lower panel. Marker sizes are indicated on the right side of each panel.

**Abbreviations:** M, marker; positive control, construct used for homologous recombination; (-) negative control, PCR without template DNA; WT, wild-type *Xtc* NXtc01; Δ*tal1*, NXtc01 *tal1* deletion mutant; Δ*tal2*, NXtc01 *tal2* deletion mutant; TF, NXtc01 *tal*-free mutant; PHZW-*tal1*, NXtc01 TF with *tal1* *in trans*; PHZW-*tal2*, NXtc01 TF with *tal2 in trans*; Δ*hrcC*, NXtc01 *hrcC* deletion mutant; and Δ*hrcC*/*hrcC*, NXtc01 Δ*hrcC* containing *hrcC in trans*.


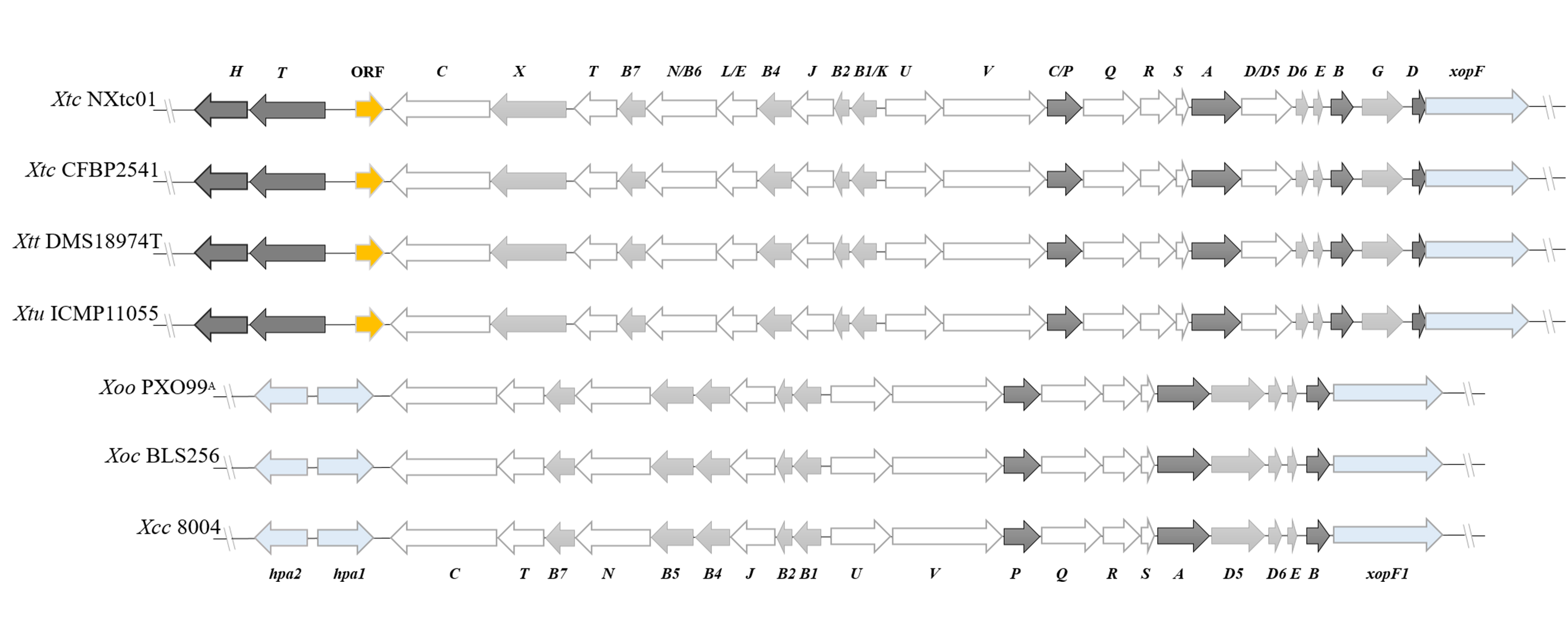


**Figure S2.** Comparison of the T3SS gene clusters in *X. translucens* pv. *cerealis* Nxtc01, *X. translucens* pv. *cerealis* CFBP 2541 (Pesce et al., 2015)*,* *X. translucens* pv. *undulosa* ICMP11055 (Falahi Charkhabi et al., 2017), *X. translucens* pv. *translucens* DSM 18974T (Jaenicke et al., 2016), *X. campestris* pv. *campestris* 8004 (Qian et al., 2005), *X. oryzae* pv. *oryzae* PX099^A^ (Salzberg et al., 2008) and *X. oryzae* pv. *oryzicola* BLS256 (Bogdanove et al., 2011). The *hrc*, *hrp*, *hpa*, T3Es and hypothetical genes are represented by white, light gray, dark gray, light blue and gold arrows, respectively; arrowheads show the transcriptional orientation. Distance between operons are not drawn to scale.

**REFERENCES**

Bogdanove, A.J., Koebnik, R., Lu, H., Furutani, A., Angiuoli, S.V., Patil, P.B., et al. (2011). Two new complete genome sequences offer insight into host and tissue specificity of plant pathogenic *Xanthomonas* spp. *Journal of bacteriology***,** JB. 05262-05211.

Falahi Charkhabi, N., Booher, N.J., Peng, Z., Wang, L., Rahimian, H., Shams-Bakhsh, M., et al. (2017). Complete genome sequencing and targeted mutagenesis reveal virulence contributions of Tal2 and Tal4b of *Xanthomonas translucens* pv. *undulosa* ICMP11055 in bacterial leaf streak of wheat. *Frontiers in microbiology* 8**,** 1488.

Hopkins, C.M., White, F., Choi, S., Guo, A., and Leach, J. (1992). Identification of a family of avirulence genes from Xanthomonas oryzae pv. oryzae. *Mol Plant Microbe Interact* 5(6)**,** 451-459.

Jaenicke, S., Bunk, B., Wibberg, D., Spröer, C., Hersemann, L., Blom, J., et al. (2016). Complete genome sequence of the barley pathogen *Xanthomonas translucens* pv. *translucens* DSM 18974T (ATCC 19319T). *Genome announcements* 4(6)**,** e01334-01316.

Pesce, C., Bolot, S., Cunnac, S., Portier, P., Fischer-Le Saux, M., Jacques, M.-A., et al. (2015). High-quality draft genome sequence of the *Xanthomonas translucens* pv. *cerealis* pathotype strain CFBP 2541. *Genome announcements* 3(1)**,** e01574-01514.

Qian, W., Jia, Y., Ren, S.-X., He, Y.-Q., Feng, J.-X., Lu, L.-F., et al. (2005). Comparative and functional genomic analyses of the pathogenicity of phytopathogen *Xanthomonas campestris* pv. *campestris*. *Genome research* 15(6)**,** 757-767.

Salzberg, S.L., Sommer, D.D., Schatz, M.C., Phillippy, A.M., Rabinowicz, P.D., Tsuge, S., et al. (2008). Genome sequence and rapid evolution of the rice pathogen *Xanthomonas oryzae* pv. *oryzae* PXO99A. *BMC genomics* 9(1)**,** 204.

Yang, B., Zhu, W., Johnson, L.B., and White, F.F. (2000). The virulence factor AvrXa7 of *Xanthomonas oryzae* pv. *oryzae* is a type III secretion pathway-dependent nuclear-localized double-stranded DNA-binding protein. *Proceedings of the National Academy of Sciences* 97(17)**,** 9807-9812.

Zou, L.-F., Li, Y.-R., and Chen, G.-Y. (2011). A non-marker mutagenesis strategy to generate poly-*hrp* gene mutants in the rice pathogen *Xanthomonas oryzae* pv. *oryzicola*. *Agricultural Sciences in China* 10(8)**,** 1139-1150.
